# How well do public sector primary care providers function as medical generalists in Cape Town: a descriptive survey

**DOI:** 10.1186/s12875-018-0802-x

**Published:** 2018-07-19

**Authors:** Renaldo Christoffels, Bob Mash

**Affiliations:** Division of Family Medicine and Primary Care, Stellenbosch University, Box 241, Cape Town, 8000 South Africa

**Keywords:** Primary care, Primary health care, Nurse practitioners, General practitioners, Consultation, Communication, Person centredness, Medical generalism, South Africa

## Abstract

**Background:**

Effective primary health care requires a workforce of competent medical generalists. In South Africa nurses are the main primary care providers, supported by doctors. Medical generalists should practice person-centred care for patients of all ages, with a wide variety of undifferentiated conditions and should support continuity and co-ordination of care. The aim of this study was to assess the ability of primary care providers to function as medical generalists in the Tygerberg sub-district of the Cape Town Metropole.

**Methods:**

A randomly selected adult consultation was audio-recorded from each primary care provider in the sub-district. A validated local assessment tool based on the Calgary-Cambridge guide was used to score 16 skills from each consultation. Consultations were also coded for reasons for encounter, diagnoses and complexity. The coders inter- and intra-rater reliability was evaluated. Analysis described the consultation skills and compared doctors with nurses.

**Results:**

45 practitioners participated (response rate 85%) with 20 nurses and 25 doctors. Nurses were older and more experienced than the doctors. Doctors saw more complicated patients. Good inter- and intra-rater reliability was shown for the coder with an intra-class correlation coefficient of 0.84 (95% CI 0.045–0.996) and 0.99 (95% CI 0.984–0.998) respectively. The overall median consultation score was 25.0% (IQR 18.8–34.4). The median consultation score for nurses was 21.6% (95% CL 16.7–28.1) and for doctors was 26.7% (95% CL 23.3–34.4) (*p* = 0.17). There was no difference in score with the complexity of the consultation. Ten of the 16 skills were not performed in more than half of the consultations. Six of the 16 skills were partly or fully performed in more than half of the consultations and these included the more biomedical skills.

**Conclusion:**

Practitioners did not demonstrate a person-centred approach to the consultation and lacked many of the skills required of a medical generalist. Doctors and nurses were not significantly different. Improving medical generalism may require attention to how access to care is organised as well as to training programmes.

## Background

Effective primary health care is an essential part of any successful health system and strengthening primary health care is a priority in South Africa especially with the huge burden of disease [1, 2]. South Africa’s vision of universal health coverage and national health insurance requires strong primary health care as a prerequisite [3]. According to the World Health Organization (WHO) one of the key reforms required of primary health care is to become more people-centred and to move away from a focus on selected diseases and vertical programmes [4]. Putting people first requires a primary care workforce that focuses on people’s health needs, is based on enduring personal relationships, is characterised by comprehensive, continuous and person-centred care, and is orientated towards tackling the underlying determinants of ill-health in a collaborative manner [5, 6].

In South Africa clinical nurse practitioners (CNPs) became the main primary care providers (PCPs) because of the shortage of doctors [7] and in order to reduce healthcare costs [8]. The adoption of nurses as the main PCP also necessitated a change in their scope of practice to be able to diagnose and prescribe. Primary care facilities include community health centres and clinics. Community health centres are larger facilities in metropolitan areas or towns and have a broader range of services offered by a multidisciplinary team that includes CNPs and doctors. Clinics are smaller facilities where services are offered by CNPs, sometimes with support from visiting doctors [9]. Pressure is placed on primary care to be comprehensive and to decrease referrals to the referral hospitals. In South Africa therefore the main medical generalist is a nurse supported by doctors.

The Royal College of General Practitioners defines medical generalism as an approach to the delivery of health care, be it to individuals, families, groups or communities, which is characterised by “whole person medicine” [10]. This broadly implies seeing a patient as a whole in the context of his or her family and community, being able to deal with undifferentiated symptoms and illness, providing a platform for continuity and coordination of care and the ability to form a collaborative relationship with both the patient and other health care providers to foster comprehensive management [11–13]. Effective communication skills are at the heart of effective generalism and the generalist must have the skills to manage these often complex consultations [14]. Direct links exist between effective communication and better health outcomes, symptom relief, reduced psychological distress, improved adherence to medication, increased patient satisfaction and less litigation [15, 16]. The principles of information sharing and concordant decision making between practitioner and patient also leads to a more effective consultation, further strengthens the therapeutic environment, and assists in providing continuity of care [13, 14]. Any health care worker that wants to function as a medical generalist, therefore, must possess and practice these capabilities [17].

Many low and middle income countries rely on nurses or mid-level doctors to provide primary care and the question therefore arises as to whether they are adequately prepared as medical generalists. Most of the evidence available is from high income countries, is qualitative and does not distinguish between CNPs working independently versus as an adjunct to the doctor. The evidence, however, suggests that patients may prefer to see a doctor if given a choice and nurses may be less prepared to offer a patient centred approach [18]. However, both doctors and nurses may provide technically competent clinical care in terms of exploring symptoms, giving acceptable advice and providing ample explanation of tests and medical terms [18]. Outcomes of care as measured by physical function, general health and vitality, social function, mental health and emotional welfare may also be similar regardless of whether care is received from a CNP or doctor [19]. In some instances CNPs had longer consultations, requested more special investigations and were less capable of providing chronic care, but had better record keeping than doctors and scored higher on amount of advice given [20, 21].

This study will add to the evidence base from a middle income country in an African setting and investigate the extent to which PCPs in public sector primary care display the attributes of a medical generalist. The findings should provide insight into the training and continuing professional development of CNPs and medical officers (MOs) functioning within a primary care team. The aim of the study was to assess the ability of PCPs to function as medical generalists in the Tygerberg sub-district of the Cape Town Metropole.

## Methods

### Study design

The study was a descriptive survey of PCPs using indirect observation of the consultation and an assessment tool.

### Setting

Cape Town has a population of 4 million people and approximately 80% are dependent on the public health services. The city is divided into eight sub-districts and this study was based in the Tygerberg sub-district, which has 10 community health centres. Two of these facilities provide 24 h emergency care whilst the others are only functioning during office hours. Three facilities also have a midwife obstetric unit providing uncomplicated obstetric care. All facilities provide emergency care, chronic care for non-communicable diseases, HIV and TB, antenatal care and integrated management of childhood illnesses.

Three facilities have a family physician (specialist in family medicine) and other specialities provide outreach via their registrars. Nursing staff consist of general registered nurses, advanced midwives, CNPs, advanced psychiatric nurses, and nurses trained in initiating antiretroviral treatment. Medical staff include established medical officers, community service medical officers and interns. The reception or triage staff allocate patients to doctors or CNPs according to prior appointments or the complexity of the problem, as doctors are meant to see more complicated patients. Patients may also be referred by the CNPs to the doctors if they need help or the guidelines require a doctor’s involvement in the management.

### Study population

The study intended to include all 53 PCPs that were consulting adults in the sub-district’s health centres and required a participation rate of at least 70% to be representative. As all PCPs in the sub-district were invited to participate there was no need to sample or select.

### Data collection

A single audio recording was made of a consultation from each PCP who gave consent. Each patient, aged 18 years and above, was randomly selected from the pool of patients waiting to see the specific PCP using a random number generator smartphone application. If consent was granted by the selected patient, then the consultation with the PCP was recorded. If the selected patient declined participation in the study, another patient was chosen with the same randomisation process. The randomisation process ensured that a range of typical patients were selected. Patients could consult in either English or Afrikaans the predominate languages in the communities served.

The Stellenbosch University Observation Tool was used for assessing the consultation. This tool is based on the Calgary-Cambridge guide to the consultation, which summarises the international evidence base for consultation skills required by medical generalists [22, 23]. Its content and construct have been validated previously by experts within the Division of Family Medicine and Primary Care. The tool has been published and is used nationally for the assessment of registrars in family medicine in all nine training programmes [24–26]. The Calgary-Cambridge guide has been shown to have reasonable score distribution with no points in the extremes, a good test-retest reliability and low inter-rater variability due to its check point system [11, 27].

The tool evaluated 16 different consultation skills (Table 1) as “not done” (score = 0), “partially done” (score = 1) or “fully done” (score = 2). Each item could also be assessed as “not applicable” to the specific consultation.

**Table 1:** Skills assessed in the observation tool

1. Makes appropriate greeting / introduction and demonstrates interest and respect	
2. Identifies and confirms the patient’s problem list or issues	
3. Encourages patient’s contribution / story	
4. Makes an attempt to understand the patient’s perspective	
5. Thinks family, and obtains relevant family, social and occupational information	
6. Obtains sufficient information to ensure no serious condition is likely to be missed	
7. Appears to make a clinically appropriate working diagnosis	
8. There is a clear explanation of the diagnosis and management plan	
9. Gives patient an opportunity to ask for other information and / or seeks to confirm patient’s understanding	
10. The explanation takes account of and relates to the patient’s perspective	
11. Involves the patient where appropriate in decision making	
12. Chooses an appropriate management plan	
13. Show a commitment to co-ordination of care	
14. Shows a commitment to continuity of care	
15. Closes consultation successfully	
16. Provides appropriate safety netting for the patient	

The assessment tool was adapted by the addition of two items to assess continuity and co-ordination of care as these were part of the definition of medical generalism. The definition of these concepts were also informed by the Primary Care Assessment Tool [28], which is another validated tool for assessing core dimensions of primary care (although not in a recorded consultation). Any statement that the healthcare worker made that indicated a commitment to informational continuity received a “partially done” score, while any statement that demonstrated a commitment to relational continuity received a “fully done” score. Any statement that the healthcare worker made which attempted to co-ordinate care between people in the facility received a “partially done” score, while any statement that indicated a commitment to co-ordinate care between external agencies in the community or the next level of care (e.g. advocating for the patient by telephone to the referral centre or local non-government organisation) received a “fully done” score. If continuity or co-ordination of care was not required, then this item was scored as “not applicable”.

Items 6, 7 and 12 were guided by the Practical Approach to Care Kit guidelines for consultation with adults in primary care, which is an evidence-based and integrated guideline for the management of common symptoms and chronic conditions in the Western Cape [29–31]. Scores were awarded on how completely the algorithm was followed; 2 was given if 75% or more of the content in the assessment or management algorithms were followed, 1 if between 50 and 74% of the content was followed and 0 if it was less than 50%.

The reasons for encounter and the diagnoses made in each consultation were coded using the International Classification of Primary Care [32]. Consultations were grouped in classes of different complexities based on the number of reason for encounter and the number of diagnoses involved in the consultation with low complexity being 1 to 2 reasons for encounter or with 1 diagnosis involved, moderate complexity 3 to 4 reasons for encounters or 2 diagnoses and high complexity having 5 or more reasons for encounter or 3 or more diagnoses [33, 34].

### Data analysis

All data was captured in Microsoft Excel and checked for errors. Data was analysed with the help of a biostatistician from Stellenbosch University’s Faculty of Medicine and Health Sciences, Biostatistics Unit, using the Statistical Package for Social Sciences software, version 24 (IBM Corp. Released 2015. IBM SPSS Statistics for Windows, Version 24.0. Armonk, NY: IBM Corp.).

Three randomly selected recordings were graded by three assessors (the researcher, an academic CNP and a family physician who were all familiar with the tool) to ensure that the primary rater had an acceptable level of reliability. For this, the Kappa value of each variable and total was calculated using Fleiss-Kappa [35, 36]. An Intraclass Correlation Coefficient was calculated to determine the level of reliability with a ratio of < 0.40 seen as poor, 0.60 to 0.74 as good and 0.75 to 1.00 as excellent [37]. The primary rater alone then re-assessed 15 randomly selected consultations four weeks after the initial assessment to determine intra-rater reliability. The Cohen-Kappa values were calculated for each individual variable and an Intraclass Correlation Coefficient was calculated to determine intra-rater reliability.

Descriptive statistics used means and standard deviations or medians and interquartile ranges for continuous data, depending on its distribution, or frequencies and percentages for categorical data.

Inferential statistics were used to compare the CNPs and MOs. The Pearson’s Chi-Square test was used to compare categorical variables between independent groups and the Mann Whitney U-test to compare median scores between practitioners (binary categories) and the Kruskal-Wallis test to compare median scores between different levels of complexity in the consultation (multiple categories). A 0.05 level of statistical significance was used.

## Results

### Profile of participants

Altogether 45 health workers were included, which gave a response rate of 45/53 (85%). Of these participants 20 were CNPs (19 females, 1 male) and 25 were MOs (19 females, 7 male). Table 2 presents their characteristics and shows that the medical officers were significantly younger and less experienced.

**Table 2:** Profile of participants

Characteristics	Clinical nurse practitioners Mean (SD)	Medical officers Mean (SD)
Age (years)	45.7 (8.5)	34.7 (10.1)
Years since qualifying as professional nurse or doctor	20.6 (8.7)	10.5 (9.3)
Years in primary care as a CNP or MO	11.8 (6.2)	5.1 (4.9)

### Rater reliability

Good inter-rater reliability was shown with an intra-class correlation coefficient for the overall assessment score of 0.84 (95% CI 0.045–0.996). High intra-rater reliability was also shown with an intra-class correlation coefficient of 0.99 (95% CI 0.984–0.998).

### Types of consultations

Table 3 shows the complexity of the cases seen by the CNPs and MOs. As expected MOs saw more complex cases than the CNPs, although overall there was a good spread of complexity across the sample. The mean consultation time was 14 min with the shortest consultation being 3 min and the longest 46 min. Table 4 shows the top 10 reasons for encounter and diagnoses involved in consultations by the PCPs.

**Table 3:** Complexity of consultations

Complexity	All N = 45 n (%)	CNPs *N* = 20 n (%)	MOs *N* = 25 n (%)
High	17 (37.8)	4 (20.0)	13 (52.0)
Moderate	13 (28.9)	8 (40.0)	5 (20.0)
Low	15 (33.3)	8 (40.0)	7 (28.0)

*CNP* Clinical nurse practitioners, *MO* Medical officers

**Table 4:** Top 10 reasons for encounter and diagnoses

	Reason for encounter (*N* = 102)	n (%)		Diagnosis (*N* = 93)	n (%)
1	Follow up appointment	14 (13.7)	1	Hypertension	14 (15.1)
2	Cough	8 (7.8)	2	Osteoarthritis	9 (9.7)
3	Back pain	7 (6.9)	3	Respiratory infection	8 (8.6)
4	Abdominal pain	6 (5.9)	4	HIV	8 (8.6)
5	Headache	6 (5.9)	5	Diabetes	6 (6.6)
6	Chest pain	5 (4.9)	6	Soft tissue injury	5 (5.4)
7	Dyspnoea	5 (4.9)	7	Urinary tract infection	4 (4.3)
8	Fatigue	4 (3.9)	8	Dyslipidaemia	4 (4.3)
9	Rash	4 (3.9)	9	Cardiac failure	3 (3.2)
10	Peripheral oedema	3 (2.9)	10	Epilepsy	3 (3.2)
11	Seizures	3 (2.9)	11	Eczema	3 (3.2)

### Evaluation of consultation skills

**Fig. 1:**
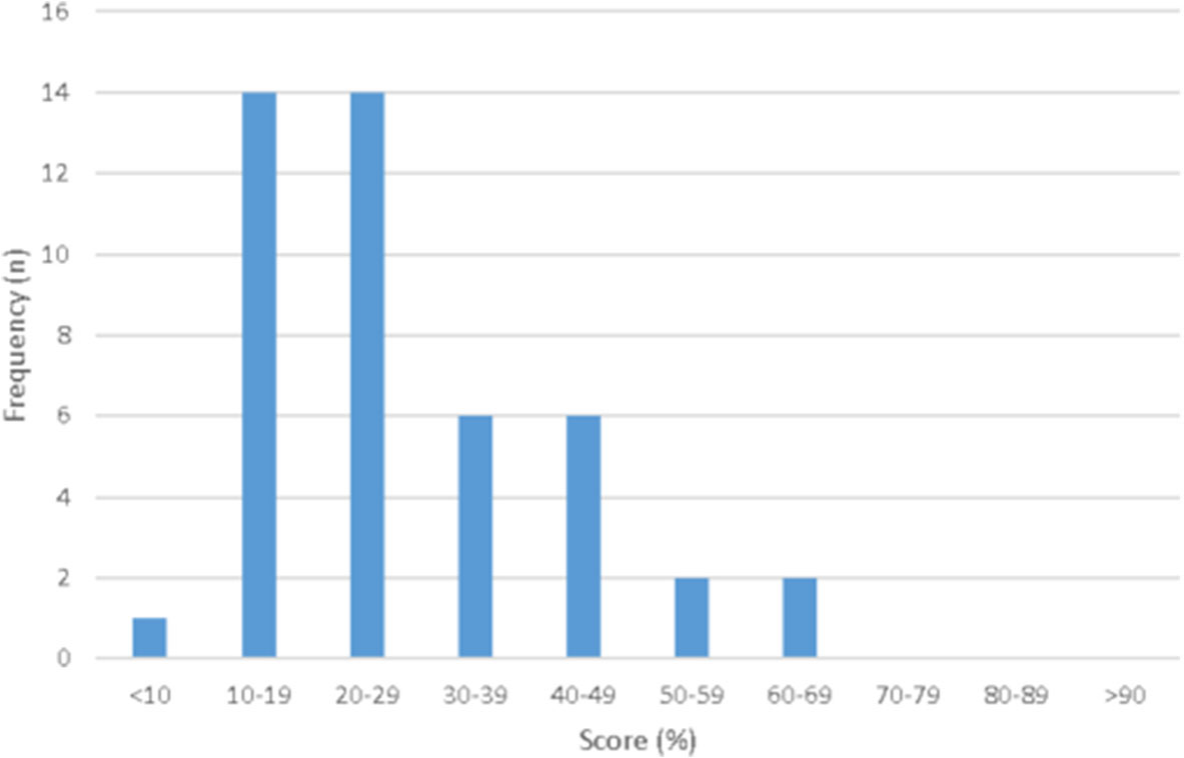
Distribution of consultation scores (*N* = 45)

Figure 1 shows the distribution of total scores as a percentage (out of maximum possible score of 32). The median score was 8.0 (IQR 6.0–11.0) and median percentage was 25.0% (IQR 18.8–34.4). The median percentage score for CNPs was 21.6% (95% CL 16.7–28.1) and for MOs was 26.7% (95% CL 23.3–34.4) (*p* = 0.17). The median percentage scores obtained for different levels of complexity in the consultation were 28.1% (95% CL 18.8–40.0) for high complexity, 23.3% (95% CL 15.6–34.4) for moderate complexity and 23.3% (95% CL 20.0–28.1) for low complexity (*p* = 0.609).

Table 5 shows how all participants performed for each skill. Ten of the 16 skills were not performed in more than half of the consultations. These missing skills were across the whole consultation and were the more patient-centred skills of building rapport, attending to the person’s perspective and context, ensuring they understood what was said and enabling shared decision making. There was little commitment to continuity of care and to safety netting for the patient. Six of the 16 skills were partly or fully performed in more than half of the consultations and these included the more practitioner-centred and biomedical skills such as collecting sufficient medical information, making an appropriate diagnosis (no diagnosis was needed in 15 consultations) or management plan and communicating these to the patients. There was some commitment to co-ordinating care.

**Table 5:** Summary of performance for each skill

Consultation skill	Not done n (%)	Partially done n (%)	Fully done n (%)
1. Makes appropriate greeting / introduction and demonstrates interest and respect	29 (64.4)	7 (15.6)	9 (20.0)
2. Identifies and confirms the patient’s problem list or issues	38 (84.4)	4 (8.9)	3 (6.7)
3. Encourages patient’s contribution / story	26 (57.8)	13 (28.9)	6 (13.3)
4. Makes an attempt to understand the patient’s perspective	40 (88.9)	3 (6.7)	2 (4.4)
5. Thinks family, and obtains relevant family, social and occupational information	36 (80.0)	8 (17.8)	1 (2.2)
6. Obtains sufficient information to ensure no serious condition is likely to be missed	13 (28.9)	20 (44.4)	12 (26.7)
7. Appears to make a clinically appropriate working diagnosis	9 (20.0)	11 (24.4)	10 (22.2)
8. There is a clear explanation of the diagnosis and management plan	13 (30.2)	20 (46.5)	10 (23.3)
9. Gives patient an opportunity to ask for other information and / or seeks to confirm patient’s understanding	34 (75.6)	7 (15.6)	4 (8.9)
10. The explanation takes account of and relates to the patient’s perspective	42 (93.3)	1 (2.2)	2 (4.4)
11. Involves the patient where appropriate in decision making	37 (82.2)	7 (15.6)	1 (2.2)
12. Chooses an appropriate management plan	7 (15.6)	18 (40.0)	20 (44.4)
13. Show a commitment to co-ordination of care	18 (40.0)	17 (37.8)	8 (17.8)
14. Shows a commitment to continuity of care	25 (55.6)	16 (35.6)	4 (8.9)
15. Closes consultation successfully	7 (15.6)	25 (55.6)	13 (28.9)
16. Provides appropriate safety netting for the patient	34 (75.6)	5 (11.1)	6 (13.3)

CNPs and MOs did not differ significantly in the percentage of skills that were fully done apart from for “obtaining sufficient information to ensure no serious condition was missed”, where the MOs performed better than the CNPs (CNPs 10% vs MOs 40%, *p* = 0.009).

## Discussion

### Summary of key findings

PCPs did not function well as medical generalists in the consultation and were particularly poor at being patient-centred. Nurses also struggled to obtain sufficient medical information to ensure no serious conditions were missed and this was an area where doctors performed significantly better. Nurses and doctors did not differ in any of the other consultation skills, although doctors were seeing more complex patients. Most consultations appeared to make an appropriate diagnosis and management plan. There was some commitment to co-ordinating care for patients, but little commitment to continuity of care. The findings suggest that despite person-centeredness being a key goal of the health system in the Western Cape [38, 39] there is a huge gap between aspiration and reality.

### Discussion of key findings

This gap in effective communication and lack of patient-centredness is likely to be one of the factors behind poor adherence to treatment [20, 21, 40], poor control of chronic diseases [18, 41] and less than ideal health outcomes in terms of quality of life and mortality [19, 42, 43]. It may also relate to increased litigation and reduced satisfaction with medical care [44]. The primary care system itself may be one of the modifiable factors behind the capacity of health workers to consult effectively. If one assumes that PCPs are capable of more holistic and effective consultations, they may be limited in their ability to perform by a high workload that necessitates large numbers of brief consultations on a daily basis. Many primary care facilities measure practitioners in terms of the number of patients seen and not the quality of the interaction or the outcomes. Many practitioners working under these stressful conditions suffer from burnout and depression [45] and this may also limit their ability to offer care [46].

There may, however, be a more fundamental gap in the capability of PCPs to communicate effectively as medical generalists. The training of clinical nurse practitioners (1-year Diploma) may not focus sufficiently on patient-centred consultation skills and may lack the opportunity to practice these skills and receive feedback [47]. The training of doctors (6-years Degree) may not consistently reinforce effective patient-centred communication skills and they may not see these skills modelled by other doctors in practice [48]. These skills are often developed further by postgraduate training in family medicine and primary care, yet few PCPs engage with such training and it is not compulsory or incentivised. The new national Diploma in Family Medicine aimed at primary care doctors does make consultation skills a core competency in the programme [49]. The training of family physicians also makes patient-centred communication a core competency (at Stellenbosch University the assessment tool used in this study was standardised at a pass mark of 60% for their exit examination, which is much higher than the median of 25% scored in actual primary care practice).

Continuity of care requires a longitudinal interaction with the same team of PCPs so that you develop a trusting and knowledgeable relationship [50–52]. This improves the efficiency and accuracy of care as ongoing management is based on a foundation of prior understanding and knowledge of the person [53]. A commitment to continuity of care was not found in this study and may reflect a lack of a systematic approach to enabling it. A lack of relational continuity is normative in these health centres [54]. These large urban community health centres do not register or link patients to specific practitioners and do not create practice teams with a sense of ongoing responsibility for a specific group of patients.

Doctors performed better than nurses in terms of gathering sufficient medical information and making an appropriate diagnosis, while also seeing more complex patients. Studies from other countries suggest that nurses can manage minor injuries in an emergency department, [8, 55] decreasing the overall workload and improving cost-effectiveness [56]. In primary care they have been shown to improve satisfaction of care, decrease the numbers referred to emergency departments, improve biomedical markers and health outcomes [57]. Nurses, however, in these more highly resourced settings may have had more relevant training and work more in collaboration with doctors rather than as replacements for them.

### Methodological issues and limitations

The behaviour of practitioners may have been affected by the presence of the audio-recorder. Such a Hawthorne effect, however, might be expected to lead to an extra effort to perform well, which would imply the scores might be lower in actual practice. If the practitioner was unduly anxious about being recorded this could also lead to a reduced performance. The audio-recorder was small and unobtrusive and may well have been forgotten as the consultation progressed. Non-verbal communication and medical record keeping were not observed. The assessment of some of the consultation skills, such as making an appropriate management plan or informational continuity of care, could have been enhanced by this collateral data. The medical officer pool included three people with post graduate training in Family Medicine (one family physician and 2 registrars) who scored much better than their peers and this would have influenced the results. Eight practitioners refused informed consent, although it is unlikely that the overall results would have been significantly different if they were included. The PCPs included in the study are typical of such practitioners in the Western Cape, although one cannot claim they are representative of PCPs throughout South Africa.

### Recommendations

Although not measured directly in this study it is clear that enabling patient-centred primary care may require managers to consider the availability of sufficient human and other resources as well as the way access is organised (e.g. appointment systems, opening times, patient flow) to ensure a reasonable consultative workload on each practitioner and the potential to offer more holistic care [58].

Pre-service training programmes for CNPs and MOs may need to give more attention to the development of patient-centred communication skills. Training needs to include theory, modelling and simulated practice, to be formally assessed and reinforced through the curriculum [47]. Thought should also be given to in-service training for existing CNPs and MOs in the form of short courses or post graduate Diplomas.

Clinical governance activities should also prioritise the acquisition of these skills and support training opportunities, quality improvement cycles and routine indicators that support the development of patient-centred communication skills.

The primary care system needs to support the development of a commitment to continuity of care by creating practice teams that take responsibility for a defined group of patients. Such an approach may dovetail with recent interest in community orientated primary care that links specific groups of households to community health workers and through them to specific health facilities and PCPs.

Comparative research could be done in rural areas, other provinces or in private general practice.

## Conclusions

PCPs did not demonstrate a person-centred approach to the consultation and lacked many of the skills required of a medical generalist. Primary care doctors, mostly without postgraduate training, and clinical nurse practitioners were not significantly different, although doctors did collect more essential medical information and saw more complex patients. Most consultations appeared to make an appropriate diagnosis and management plan. There was little commitment to continuity of care and moderate commitment to co-ordination of care. Improving person-centredness and medical generalism may require attention to how access to care is organised as well as to pre-service, postgraduate and in-service training programmes.

## Abbreviations

CNP: Clinical nurse practitioner
HIV: Human immunodeficiency virus
MO: Medical officer
PCP: Primary care provider
TB: Tuberculosis
WHO: World Health Organization
